# Nivolumab-induced thyroid dysfunction lacking antithyroid antibody is frequently evoked in Japanese patients with malignant melanoma

**DOI:** 10.1186/s12902-018-0267-x

**Published:** 2018-06-08

**Authors:** Seiichi Yano, Kenji Ashida, Hiromi Nagata, Kenji Ohe, Naoko Wada, Yukina Takeichi, Yuki Hanada, Yuta Ibayashi, Lixiang Wang, Shohei Sakamoto, Ryuichi Sakamoto, Hiroshi Uchi, Motoaki Shiratsuchi, Masutaka Furue, Masatoshi Nomura, Yoshihiro Ogawa

**Affiliations:** 1 Department of Medicine and Bioregulatory Science, Graduate School Of Medical Sciences, Kyushu University, 3-1-1 Maidashi, Higashi-Ku, Fukuoka, 812-8582 Japan; 2 Faculty of Pharmaceutical Sciences, Fukuoka University, 8-19-1 Nanakuma, Jonan-ku, Fukuoka, 814-0180 Japan; 3 Department of Dermatology, Kyushu University Hospital, 3-1-1 Maidashi, Higashi-ku, Fukuoka, 812-8582 Japan; 4 Division of Endocrinology and Metabolism, Department of Internal Medicine, Kurume University School of Medicine, 67 Asahi-machi, Kurume, Fukuoka, 830-0011 Japan

**Keywords:** Thyroid, Nivolumab, Thyroiditis, Hypothyroidism, Programmed cell death − 1

## Abstract

**Background:**

Nivolumab, an anti-programmed cell death-1 monoclonal antibody, has improved the survival of patients with malignant melanoma. Despite its efficacy, nivolumab inconsistently induces thyroid dysfunction as an immune-related adverse event (irAE). This study aimed to evaluate nivolumab-induced thyroid dysfunction to determine the risks and mechanisms of thyroid irAEs.

**Methods:**

After excluding 10 patients, data of 24 patients with malignant melanoma (aged 17–85 years; 54% female) were retrospectively analyzed.

**Results:**

Thyroid irAEs were observed in seven patients (29%). Three patients had hypothyroidism after preceding transient thyrotoxicosis, and the other four patients had hypothyroidism without thyrotoxicosis. Levothyroxine-Na replacement was required in three patients. Antithyroid antibody (ATA) titer was elevated in one of four assessable patients. The average (±SD) time to onset of thyroid irAE was 33.6 (±21.9) weeks. The administration period of nivolumab was longer in patients with thyroid irAEs than in those without thyroid irAEs (*P* < 0.01). There were no significant differences between patients with and without thyroid irAEs regarding age, sex, tumor stage, response to nivolumab therapy, baseline thyroid function, antithyroid peroxidase antibody (anti-TPO Ab) and antithyroglobulin antibody (anti-Tg Ab).

**Conclusions:**

Thyroid dysfunction was a common irAE of nivolumab in malignant melanoma. Neither anti-TPO Ab nor anti-Tg Ab was associated with the risk for nivolumab-induced thyroid dysfunction. A conventional ATA-independent mechanism might be involved in thyroid irAEs. Further studies are required to clarify the mechanism and identify the predictive factors of thyroid irAEs.

## Background

Nivolumab, an anti-programmed cell death-1 (PD-1) antibody, has emerged as a breakthrough medication for several advanced malignancies [1, 2]; however, it frequently induces thyroid dysfunction [3, 4], possibly by evoked autoimmunity, similar to other immune-related adverse events (irAEs) [5–10].

Although thyroid dysfunction with thyroiditis is the most frequently reported nivolumab-induced endocrinopathy [1, 2], the reason why pathological autoimmunity of the thyroid is evoked by nivolumab remains unknown. In addition, autoimmune thyroid disease is common, but the mechanism of its development is not fully understood.

Here, we report the clinical characteristics and parameters of nivolumab-induced thyroid dysfunction in patients with melanoma. Thirty-four patients who were treated with nivolumab at our hospital were examined. This study aimed to evaluate nivolumab-induced thyroid dysfunction to determine the risks and mechanisms of thyroid irAEs**.**

We should take into consideration thyroid irAEs for successful treatment with this beneficial medication because we observed the occurrence of nivolumab-induced thyroid dysfunction more frequently in our study than in previous reports [10, 11]. Furthermore, regarding nivolumab-induced thyroiditis without relation to conventional antithyroid antibody (ATA), the novel subtype of PD-1-related autoimmune thyroiditis without both antithyroid peroxidase antibody (anti-TPO Ab) and antithyroglobulin antibody (anti-Tg Ab) observed in this study might give us a novel clue in deciphering the mechanism underlying autoimmune thyroiditis.

## Methods

**Fig. 1:**
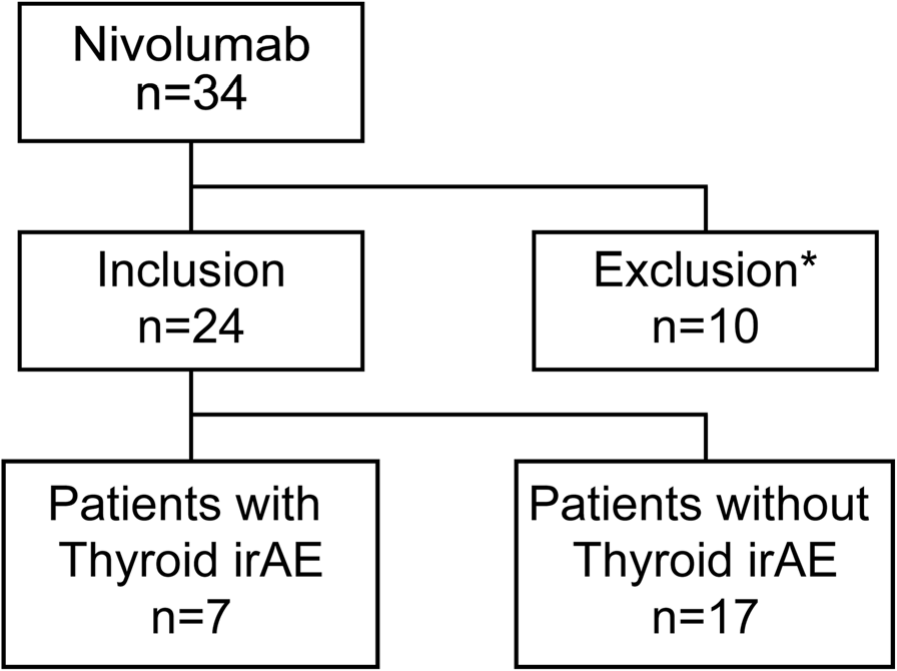
Schematic overview of nivolumab-treated patients with malignant melanoma The inclusion criteria and numbers of each group are described. irAE, immune-related adverse event. *Patients with current active thyroid dysfunction and without evaluation of thyroid function were excluded

The study retrospectively recruited all of 34 patients with advanced malignant melanoma who were treated with nivolumab (Bristol-Myers Squibb, Princeton, NJ, USA) at Kyusyu University Hospital from September 1, 2014 to September 30, 2016 (Fig. 1). The patients were intravenously administered 3 mg/kg nivolumab and evaluated thyroid function every 3 weeks. Patient characteristics such as sex, age, tumor stage, history of thyroid disorder, response to nivolumab therapy, duration of nivolumab therapy, thyroid function, and ATA were retrieved from medical records. In patients with thyroid irAEs, ATA titers were assessed at the time when thyroid dysfunction was detected (Table 1).

**Table 1:** Patient characteristics

		Total *n* = 24	Thyroid irAE *n* = 7	No Thyroid irAE *n* = 17	*P Value *
Sex	Male	11	2	9	0.39
	Female	13	5	8	
Age	Median	61.5 ± 17.4	57.0 ± 19.0	63.0 ± 17.1	0.57
	Range	(50–75)	(37–76)	(51–75)	
	≤40 ≤ 40	3	2	1	
	> 40	21	5	16	
Tumor staging	II	1	0	1	0.91
	III	2	1	1	
	IV	21	6	15	
Metastasis stage	M1a	2	0	2	0.7
	M1b	9	3	6	
	M1c	10	3	7	
Response to nivolumab	PR	2	1	1	0.68
	SD	10	4	6	
	PD	6	2	4	
	NA	6	0	6	
History of previous thyroid disorder	YES	2	1	1	0.51
	NO	22	6	16	
Administration duration^b^, week		27.5 ± 25.4	54.4 ± 24.1	16.4 ± 17.9	**0.0045**
Administration duration including rest period^b^, week		32.1 ± 31.4	59.9 ± 26.8	20.6 ± 26.2	**0.0075**
Number of administration^b^		9.5 ± 7.2	16.7 ± 5.4	6.6 ± 5.7	**0.0016**
TSH^b^, μU/mL		1.94 ± 1.30	2.22 ± 1.21	1.82 ± 1.35	0.51
fT4^b^, ng/dL		1.25 ± 0.19	1.19 ± 0.13	1.27 ± 0.21	0.3
fT3^b^, pg/mL, n		2.93 ± 0.48, 11	3.20 ± 0.23, 3	2.80 ± 0.51, 8	0.063
Elevated anti-Tg Ab, case/total (%)		2/17 (12%)	1/4 (25%)	1/13 (8%)	0.47
Elevated anti-TPO Ab, case/total (%)		0/17 (0%)	0/4 (0%)	0/13 (0%)	1.0
Elevated TRAb, case/total		1/4	0/0	1/4	NA

Reference laboratory values are as follows: TSH, 0.27–4.20 μU/L; fT4, 1.0–1.8 ng/dL; fT3, 2.2–4.4 pg/mL; anti-TPO Ab, < 30 IU/L; anti-Tg Ab, < 30 IU/L; TRAb, < 2 IU/L.^a^Values are presented as median ± standard deviation (SD). ^b^Values are presented as mean ± SD and represent administration duration, including rest period; number of administrations; and TSH, fT4, and fT3 levels. Significant differences are indicated in bold font

Abbreviations: *irAEs*, immune-related adverse events; *fT4* free thyroxine, *fT3* free triiodothyronine, *TSH* thyroid-stimulating hormone, *anti-TPO Ab* antithyroid peroxidase antibody, *anti-Tg Ab* antithyroglobulin antibody, *TRAb* TSH receptor antibody, *PR* partial response, *SD* stable disease, *PD* progressive disease; NA, not applicable

At the initial nivolumab therapy, 10 patients were excluded for the following reasons: six because with their thyroid function not evaluated due to early death, one with active hyperthyroidism, two with overt hypothyroidism, and one with secondary hypothyroidism. Two patients with a history of thyroid disorder but without ATA were included because they showed normal thyroid function and did not require active therapy: one with history of hemi-thyroidectomy due to papillary thyroid carcinoma and the other with a history of Graves’ disease and having TSH receptor antibody (TRAb). None of the patients had a history of any disease that required immunomodulating agents or pretreatment with other checkpoint inhibitors such as ipilimumab, which is an anti-cytotoxic T-lymphocyte associated protein-4 antibody.

The baseline clinical features of the patients are summarized in Table 1. All the remaining 24 patients were included and evaluated for free thyroxine (fT4), free triiodothyronine (fT3), thyroid-stimulating hormone (TSH), anti-TPO Ab, and anti-Tg Ab. We defined thyrotoxicosis, hypothyroidism, and subclinical hypothyroidism according to the TSH and fT4 levels, following a previous study of irAEs [3]. Thyrotoxicosis was defined as the presence of suppressed TSH level with elevated fT4 level. Hypothyroidism was defined as the presence of elevated TSH level with decreased fT4 level. Subclinical hypothyroidism was defined as the presence of elevated TSH level with normal fT4 level. Reference range of TSH and fT4 is 0.27–4.20 μU/L and 1.0–1.8 ng/dL, respectively. Thyroid irAEs were graded according to the Common Terminology Criteria for Adverse Events version 4.03 [12]. Baseline tumor staging was according to the American Joint Committee on Cancer (AJCC) Staging Manual, seventh edition [13].

### Statistical analysis

All statistical analyses were performed using JMP® 13 (SAS Institute Inc., Cary, NC, USA). Patient, tumor, and treatment variables were compared using the Fisher’s exact (sex, history of previous thyroid disorder, and elevated antithyroid antibodies), Mann–Whitney–Wilcoxon (age, tumor staging, metastasis, and response to nivolumab), and the unpaired two-sample *t* test (baseline value of mean TSH, fT4, and fT3; period of nivolumab administration; number of administration). Significance was defined as a *P*-value < 0.05.

## Results

### Patient characteristics

There were no significant differences between patients with and without thyroid irAEs regarding sex, age, tumor stage, response to nivolumab therapy, and baseline thyroid function (Table 1). The average duration (±SD) of nivolumab administration up to the time when thyroid irAE was diagnosed was 33.6 ± 21.9 weeks. The average duration of nivolumab administration was longer in patients with thyroid irAEs than in those without thyroid irAEs (*P* < 0.01). The administration durations in patients without thyroid irAEs were all shorter than 33.6 weeks, which was the average administration period in patients with thyroid irAEs.

### Thyroid-related adverse events

**Fig. 2:**
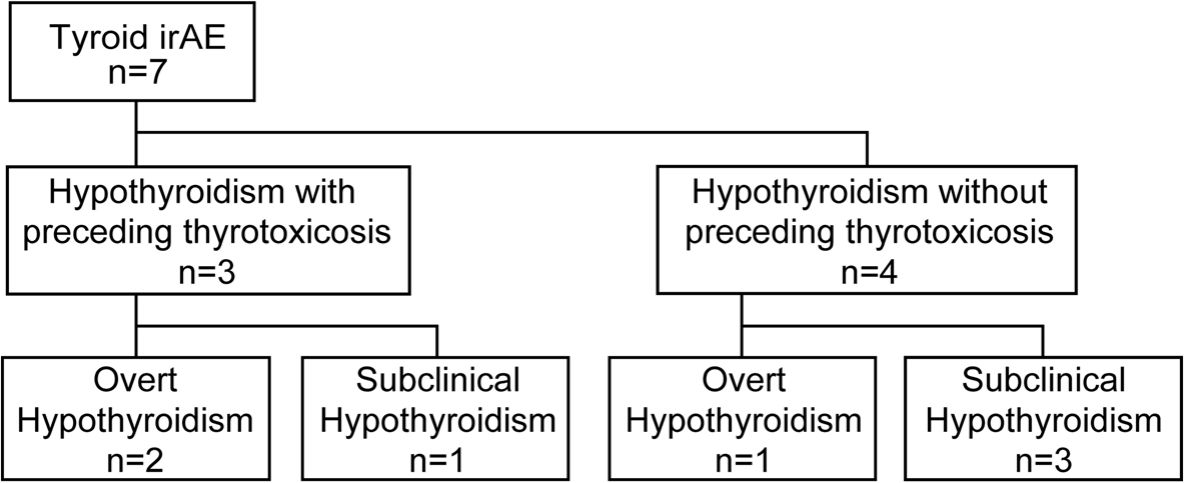
Overview of thyroid immune-related adverse events (irAEs) in patients with melanoma treated with nivolumab The continuous levothyroxine replacement was needed in two out of three hypothyroid patients who had preceding transient thyrotoxicosis and in only one out of four hypothyroid patients without preceding thyrotoxicosis. Abbreviations: irAE, immune-related adverse event

Thyroid irAEs were observed in seven of 24 patients (29%), with four having grade 1 irAEs and three having grade 2 irAEs, when we evaluated them every 3 weeks (Fig. 2). Three of the seven patients had transient thyrotoxicosis, eventually resulting in hypothyroidism. The remaining four had hypothyroidism without preceding thyrotoxicosis. Levothyroxine-Na replacement was continuously required for three of the seven patients; the thyroid function of the other four patients spontaneously resolved to normal function. Two of the three patients with overt hypothyroidism had preceding transient thyrotoxicosis.

Four patients developed secondary adrenal insufficiency, and all of them required continuous hydrocortisone replacement. One of them developed hypothyroidism 38 weeks after the start of nivolumab treatment. Another three patients developed secondary adrenal insufficiency during ipilimumab treatment after the termination of nivolumab, and two of them did not show thyroid dysfunction. The remaining patient developed hypothyroidism 5 months after the onset of secondary adrenal insufficiency, which was equivalent to 64 weeks after nivolumab administration.

### Antithyroid antibodies

ATA (anti-Tg Ab and anti-TPO Ab) titers were assessed in 17 patients (Table 1). Anti-Tg Ab titer was elevated in one of the four patients with thyroid irAEs (25%) and one of the 13 patients without thyroid irAEs (8%). Anti-TPO Ab was not detected in all 17 patients. TRAb titer was examined in four patients. Although one patient with a history of Graves’ disease had elevated TRAb titers, other three were negative. None of the four patients developed thyroid irAEs.

## Discussion

We report the clinical features of thyroid dysfunction in all patients who were treated with nivolumab for advanced malignant melanoma at our hospital. First, we found that a high proportion (29%) of patients developed nivolumab-induced thyroid irAEs. Second, ATA titers would possibly not be useful for predicting the risk for nivolumab-induced thyroid dysfunction. Third, thyroid-specific autoimmunity induced by nivolumab might be independent of its antitumor efficacy.

A higher prevalence of thyroid dysfunction in patients with advanced melanoma treated with nivolumab was observed in this study than in previous reports. Recent pooled analysis including two phase III trials of nivolumab [10] reported 4.2% of 576 patients with melanoma complicated with hypothyroidism and 2.1% with melanoma complicated with hyperthyroidism. In another study of 206 patients with metastatic melanoma [11], nivolumab induced hypothyroidism and hyperthyroidism in 4% (nine cases) and 3% (seven cases), respectively. In addition, 69% of the patients were from Europe or Canada and 31% were from Israel, Australia, or South America. Our study may indicate a difference in prevalence of nivolumab-induced thyroid dysfunction between Japan and Western countries. Another report of Japanese patients recently found that 21% (3/14) of nivolumab-treated patients with malignant melanoma developed thyroid dysfunction [14, 15]. Because that study did not refer to transient or subclinical hypothyroidism, the frequency of thyroid irAEs in Japan could be higher, as in our study.

Anti-TPO Ab and anti-Tg Ab titers would possibly not be useful for predicting the risk for nivolumab-induced thyroid dysfunction. In our study, only 25% (1/4) of patients with thyroid irAEs had elevated anti-Tg Ab titers, with none having elevated anti-TPO Ab titers (Table 1). This result suggested that at least three patients were both anti-TPO and anti-Tg Ab negative before nivolumab treatment, although anti-TPO and anti-Tg Ab have been expected to have a predictive role in thyroid irAEs [4, 14]. Elevated ATA, especially anti-TPO Ab titers were shown to correlate with the development of overt hypothyroidism [16, 17]. In a previous study, anti-TPO Ab was detected in 67% of patients with painless thyroiditis after nivolumab therapy [4]. Our current study proposed that a subtype of anti-TPO and anti-Tg Ab double-negative autoimmune thyroiditis was induced by nivolumab more frequently in Japan than in Western countries. TRAb might provide us useful information on thyroid irAEs because of recent advances in TRAb titer measurement [18–20]. Although one patient with positive TRAb did not develop thyroid dysfunction, the measurement of TRAb titers might provide us valuable information on the etiology of PD-1-induced thyroid irAEs.

Thyroid-specific autoimmunity induced by nivolumab might be independent of the antitumor efficacy of nivolumab. Some studies suggest that the occurrence of irAEs in patients treated with immune checkpoint inhibitors may be correlated with an amelioration of malignancies [14, 21, 22]. Despite these reports, we found no substantial difference in antitumor effect between patients with and without thyroid irAEs, although among seven patients with thyroid irAEs, 14% (1/7) had a clinical response compared with 6% (1/17) of patients without thyroid irAEs (Table 1).

We proposed that autoimmune thyroid dysfunction without both anti-TPO and anti-Tg Ab was induced by nivolumab. Several environmental and genetic factors have been reported as triggers of autoimmune thyroid diseases [23]. The genes responsible for autoimmune thyroid diseases could be categorized as either thyroid-specific types (e.g., thyroglobulin and TSH receptor) or immune-modulating types (e.g., Forkhead box P3 (FOXP3), CD25, CD40, CTLA-4, and HLA) [23]. FOXP3 is a crucial regulatory factor for the development and function of regulatory T cells (Treg) [24], and its deficiency suppresses the regulatory function of Treg cells [25]. Furthermore, polymorphisms of *FOXP3* play a role in genetic susceptibility to autoimmune thyroid diseases [26], and the genotype of *FOXP3* was reported to have an association with the severity of Hashimoto’s disease [27]. PD-1 is expressed on activated T cells, including Treg cells, and its expression by Treg cells is required to maintain immune tolerance [28]. Nivolumab-induced thyroid irAEs would clarify the etiology of conventional ATA-negative autoimmune thyroid diseases that cannot be diagnosed as definite Hashimoto’s thyroiditis.

PD-1 may be a key regulator for preventing a subtype of autoimmune thyroiditis, which was found in the Japanese population. The prevalence of Hashimoto’s thyroiditis correlated with the Th1 response and cellular immunity was reported to be higher in Caucasian than in Japanese, while the prevalence of Graves’ disease correlated with the Th2 response was reported higher in Japanese than in Caucasians [29–31]. As the present study indicates, nivolumab-induced thyroid irAEs might be more frequently observed in Japanese, because PD-1 blockade was reported to invert the immune response from a Th2-dominant to a Th1-dominant state [31]. PD-1 blockade with nivolumab probably induced non-conventional ATA-independent autoimmunity, particularly in Th2-dominant Japanese patients.

This study has some limitations. First, the number of patients treated with nivolumab was small; thus, studies with larger number of patients will be necessary to clarify the features of nivolumab-induced thyroid irAEs. Second, we did not evaluate ATA in all patients before the onset of irAE, and the TRAb titer was not examined in patients with thyroid irAEs. Third, treatment duration, especially that in non-thyroid irAE group, was short because the occurrence of irAEs can be delayed [2]. Finally, we could not examine the histopathological features of the affected thyroid glands of patients with thyroid irAEs. Further studies are under way to identify predictive markers and to clarify the etiology of thyroid irAEs in Japanese patients. We believe these findings will uncover a subtype of autoimmune thyroid diseases without known ATA.

## Conclusions

When advanced melanoma patients were treated with nivolumab, 29% of them developed thyroid dysfunction. Anti-TPO and anti-Tg Ab titers were not useful for predicting the risk for nivolumab-induced thyroid dysfunction. Therefore, constant monitoring and awareness of thyroid irAEs are recommended, particularly in patients undergoing long-term nivolumab therapy. Nivolumab may induce thyroid irAEs by evoking non-conventional thyroid-specific autoimmunity. Further studies are required to clarify the characteristics and predictive factors of thyroid irAEs.

## Abbreviations

Anti-Tg Ab: antithyroglobulin antibody
Anti-TPO Ab: antithyroid peroxidase antibody
ATA: antithyroid antibody
FOXP3: Forkhead box P3
fT3: free triiodothyronine
fT4: free thyroxine
irAE: immune-related adverse event
PD-1: programmed cell death-1
TRAb: TSH receptor antibody
Treg: regulatory T cells
TSH: thyroid-stimulating hormone
